# ELANE基因突变导致先天性中性粒细胞减少症1例

**DOI:** 10.3760/cma.j.issn.0253-2727.2023.09.016

**Published:** 2023-09

**Authors:** 新新 刘, 静 董, 杰 李, 庆华 刘, 红 张

**Affiliations:** 1 山东第一医科大学第二附属医院血液科，泰安 271000 Department of Hematology, the Second Affiliated Hospital of Shandong First Medical University, Taian 271000, China; 2 山东第一医科大学第二附属医院检验科，泰安 271000 Department of Clinical Laboratory, the Second Affiliated Hospital of Shandong First Medical University, Taian 271000, China

患者，男，23岁，因“反复感染伴中性粒细胞减少20余年”入院。患者出生后2周即出现脐炎，后伴支气管肺炎、口腔溃疡、肛周溃疡反复发作（每年发作10～12次），抗感染治疗后可好转。患者因反复口腔感染，牙齿逐渐脱落。曾查血常规发现WBC 4.87×10^9^/L，中性粒细胞绝对值（ANC）0.19×10^9^/L，HGB、PLT正常；当地医院考虑中性粒细胞缺乏症，间断予以G-CSF升白细胞治疗，效果不理想。1年前因化脓性阑尾炎行腹腔镜手术，术后切口迁延不愈遂就诊于我院。

查体：颈部可触及肿大淋巴结，质软。舌体溃疡，所有磨牙均缺如。右下腹可见一约1.0 cm×1.5 cm大小手术切口感染窦道。血常规：WBC 3.39×10^9^/L，ANC 0.01×10^9^/L，HGB 134 g/L，PLT 241×10^9^/L。直接抗人球蛋白试验（+）；ANA、ENA谱等常见自身抗体均阴性。鼻咽部、胸腹部CT：①口咽右后壁软组织增厚；②上颌窦炎症；③双肺慢性炎症；④脾略增大，局限膨隆；⑤回肠末端与邻近的乙状结肠肠壁增厚，伴分界不清，考虑为炎性改变，回肠-乙状结肠瘘不除外。骨髓象示粒系增生低下，偏成熟阶段粒细胞少见。染色体核型：46，XY[20]。

遗传性血液病相关基因检测：发现变异基因ELANE。全外显子组测序：发现ELANE基因杂合突变（c.197T>G）（exon2/5）（p.M66R）；患者父母及哥哥均无该基因位点变异（图1）。该位点系我国首例在先天性粒细胞减少症中发现的新的变异位点。患者最终诊断为ELANE相关的先天性中性粒细胞减少症，每10～12 d应用PEG-rhG-CSF 6 mg皮下注射，外周血ANC可维持在2.0×10^9^/L以上，且患者未再发生严重感染事件。

**图1:**
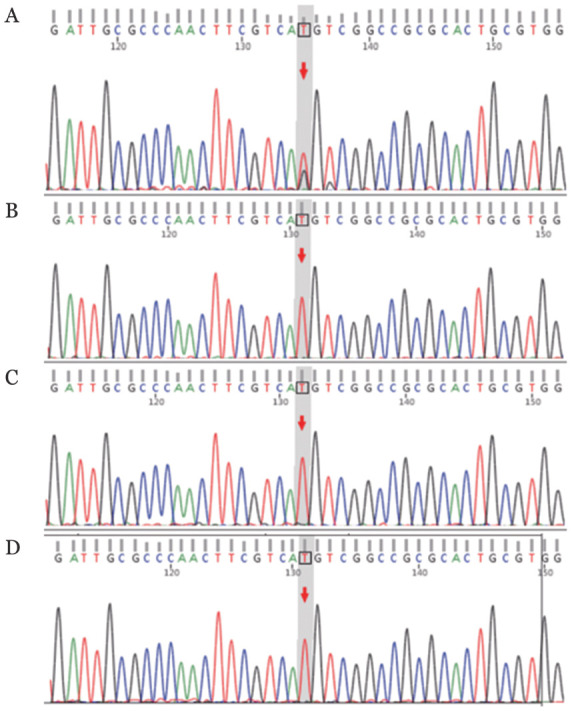
先天性中性粒细胞减少症患者及直系亲属ELANE基因测序结果 **A** 患者存在c.197T>G位点杂合变异；**B**、**C**、**D** 患者父亲、母亲、哥哥均无c.197T>G位点杂合变异

